# Experiences of patients with hypertension at primary health care in facilitating own lifestyle change of regular physical exercise

**DOI:** 10.4102/curationis.v40i1.1679

**Published:** 2017-04-26

**Authors:** Nomasonto B.D. Magobe, Marie Poggenpoel, Chris Myburgh

**Affiliations:** 1 Department of Nursing Sciences, University of Johannesburg, South Africa; 2 Department of Nursing Sciences, University of Johannesburg, South Africa; 3 Department of Education, University of Johannesburg, South Africa

## Abstract

**Background:**

Regular physical exercise is one of the lifestyle modification general measures to control the blood pressure (BP) of patients with hypertension. Globally, hypertension is considered a non-communicable disease (NCD), as well as a chronic condition of lifestyle, that contributes to the mortality rate caused by complications of cardiovascular burden of diseases. In South Africa, NCDs account for nearly 40% of adult deaths, with a high prevalence among black people in urban areas such as Soweto.

The first step in treating hypertension is lifestyle modification, referred to in this study as health-promoting lifestyle change measures. Despite the positive benefits of regular physical exercise in controlling hypertension, in 2014, only 10% of men and 18% of women with hypertension had their BP controlled to a level that would eliminate the risk of cardiovascular disease (CVD) complications.

**Objectives:**

The aim of this article is to present the experiences of patients with hypertension regarding the facilitation of their own health-promoting lifestyle change measure of regular physical exercise.

**Method:**

A qualitative, exploratory, descriptive and contextual research design was used. The accessible population of patients with hypertension at three primary health care (PHC) clinics in Soweto was targeted and purposefully sampled. Focus group and individual interviews were conducted to collect data till data saturation occurred. Tesch’s open-coding method of data analysis was used.

**Results and conclusions:**

Findings show that participants experienced poor self-care due to poor self-efficacy, demonstrated by not engaging in regular physical exercise, which in turn, resulted in uncontrolled BP and cardiovascular complications from hypertension. More should be done to educate, motivate and empower patients with the necessary knowledge, skills and the values in facilitating their own regular physical exercise in order to improve their own quality of health.

## Introduction and background

Hypertension is globally considered to be the greatest mortality risk factor for both men and women and is regarded as a modifiable risk factor of the non-communicable diseases (NCDs) of lifestyle (Moosa, Kuttschreuter & Rayner 2016; WHO 2011). A global study by Forouzanfar and his collaborators from 188 countries (Forouzanfar et al. 2015) confirmed that the global, regional and national risk factors of hypertension increased by almost 50% between 1990 and 2013. In South Africa, hypertension is also a common chronic disease of lifestyle responsible for more deaths than any other cardiovascular chronic condition (Seedat & Rayner 2012). To combat this scourge of hypertension, the South African strategic plan for the prevention of NCDs, as a 2020 target, is to reduce the prevalence of hypertension by 20% through lifestyle modification (Day et al. 2014). One of the lifestyle modification strategies is regular physical exercise. Thus, one of the strategic plans is to increase the prevalence of moderate intensive physical activity to 150 min per week by 10% for persons above the age of 18 years, as further mentioned by Day et al. (2014).

In South Africa, NCDs, including hypertension, account for 40% of adult deaths, with a higher prevalence in black South Africans living in urban and peri-urban local communities (Seedat & Rayner 2012). Soweto is an urban black-dominated residential township, which is fast becoming a city of the 21st century with shopping malls and fast-food outlets. Urban areas like Soweto also show increasing evidence of sedentary lifestyles due to people spending more time watching television (TV), working on personal computers (PC), engaging on smart phones, being occupied by social media, texting messages and playing PC or TV games.

A sedentary lifestyle in an urban area like Soweto can also be caused by modern lifestyle conveniences such as the use of elevators, escalators, including increasing number of people driving cars, a higher availability of mini-bus taxis for commuting and motor cycles belonging to bikers’ clubs, which all adds up to less physical activity as confirmed by Eating Disorders South Africa (EDSA) (2012). It is estimated that children spend 600 kilocalories per day (kcal/day) less on physical activity than children had done 50 years ago (EDSA 2012). This will, in turn, increase the number of future adults with potential NCDs such as hypertension as stated in Health24 News (2014). There is also evidence of a rapid increase in the prevalence of chronic diseases associated with lifestyle changes as mentioned by Vancampfort et al. (2017).

Literature shows that hypertension is more prevalent in urban areas than in rural areas (Addo, Smeeth & Leon 2007; Ibrahim & Damasceno 2012). There is, however, poor awareness of hypertension and also low control of blood pressure (BP) in urban areas like Soweto, as pointed out by Ibrahim and Damasceno (2012). The poor awareness and low control of hypertension are supported by the findings of a study by Tibazarwa et al. (2008), showing that Soweto residents had a poor awareness of common cardiovascular disease (CVD) modifiable risk factors such as hypertension.

Hypertension, being a lifestyle chronic condition, is by implication a lifelong condition that may not be curable. However, Smith et al. (2010) maintain that hypertension can be controlled in 50% of adults through healthy lifestyle changes. The first step in the management of hypertension is lifestyle modification (Seedat & Rayner 2012).

Lifestyle modification is a non-pharmacological treatment for the control of BP in patients with hypertension and implies a change or modification from an unhealthy to a healthy lifestyle (Seedat & Rayner 2012). The aim of lifestyle modification is to promote a patient’s general health; thus, in this study, lifestyle modification is referred to as ‘health-promoting lifestyle’, and the lifestyle modification measures are referred to as ‘health-promoting lifestyle change measures’. The main purpose, therefore, of health-promoting lifestyle measures such as regular physical exercise is to lower the BP, as well as the subsequent improvement of hypertensive patient’s quality of life (Klabunde 2014; Seedat & Rayner 2012; Stuart-Shor et al. 2012).

Literature shows that a healthy lifestyle, which includes regular physical exercise, causes the lowering of BP by 5 mmHg–6 mmHg over 5–6 years of follow-up chronic care (Mohan, Seedat & Pradeepa 2013; Seedat & Rayner 2012). Further evidence indicates a CVD mortality rate reduction of 21% when patients adhere to a healthy lifestyle (Mohan et al. 2013). Regular physical exercise, as an important health-promoting lifestyle change measure, implies regular aerobic exercise for about 10 to 30 min a day in order to improve cardiovascular health (Stuart-Shor et al. 2012) for people with or without hypertension. This then means that regular physical exercise may prevent the development of hypertension in those individuals who do not yet have hypertension, and for patients with hypertension, regular physical exercise contributes to the reduction of their BP (ESSA 2011; Stuart-Shor et al. 2012).

The first strategic objective of the National Sport and Recreation Plan (NSRP) is to improve the health and well-being of the South African population by providing mass participation opportunities through active recreation (Sport and Recreation South Africa [SRSA] 2012). The NSRP has implemented several activities in South Africa to curb the scourge of NCDs among children, who are the future adult citizens, and to also promote regular physical exercise within the entire population of South Africa. One such activity is ‘*Siyadlala*’, meaning ‘We play’ (SRSA 2012; Strydom 2013). *Siyadlala* is aimed at ‘getting the nation to play’ as implemented by the Department of Sport and Recreation (SRSA 2009, 2012). The Departments of Health and of Education, including Sports and Recreation, are all collaborating to increase the physical activity of the nation by means of the ‘*Vuka* South Africa’ meaning ‘Move for your Health South Africa’ campaign, which was launched in May 2005 (SRSA 2009, 2012; Strydom 2013). Other strategies, for example, The Charter for Physical Activity, Sport, Play and Well-Being for all Children and Youth in South Africa, and the Youth Fitness and Wellness Charter, were all initiated with a view to increase mass participation in regular physical ‘aerobic’ exercise and activity (SRSA 2012). Patients with hypertension are also encouraged to become involved in regular physical aerobic exercise or other physical activity that the primary health care (PHC) clinics in Soweto’s physiotherapy departments present. This notion is emphasised by Vancampfort et al. (2017) by stating that the efficacy and effectiveness of physical exercise in the management of chronic conditions should be prioritised at all levels of health care. The programmes promote aerobic physical exercises, which are exercises for heart and lung fitness, involving physical activities such as walking, jogging, cycling or swimming for 4–7 days a week (ESSA 2011; Stuart-Shor et al. 2012), which all promote heart and lung function and improve hypertension control.

Regular aerobic exercise also helps to maintain one’s ideal body mass. This then indicates that health-promoting lifestyle change measures such as regular physical exercise and loss of body mass complement each other. Ferris (2010) and Mohan et al. (2013) also maintain that bad health is indicated not only by illness but also by a lack of exercise and excessive body mass gain that increases the risk of chronic conditions of lifestyle, such as hypertension, diabetes mellitus and others. For patients who already have a chronic condition like hypertension, the SRSA’s 2016/17 Annual Performance Plan (APP) continues to facilitate the provision of campaigns and programmes that increase active participation leading to lifelong wellness (SRSA 2016).

## Problem statement and the research question

A healthy lifestyle is the cornerstone of the treatment and control of all hypertension, from the pre-hypertension stage to the mild, moderate and severe stages of hypertension, as confirmed by the South African National Department of Health and the Southern African Hypertension Society (SAHS) (Seedat & Rayner 2012). Health-promoting lifestyle change measures such as regular physical exercise remain to be the first step in the treatment of hypertension at all levels of health care (Seedat & Rayner 2012; Vancampfort et al. 2017).

Regular physical exercise as a health-promoting lifestyle change measure has been proven to decrease BP and enhance pharmacological or drug efficacy, resulting in the reduction of total cardiovascular risk and complications (Mohan et al. 2013; Seedat & Rayner 2012).

Despite such proven benefits of regular physical exercise on BP, patients with chronic conditions like hypertension are less likely to engage in regular physical exercise, as mentioned by Vancampfort et al. (2017), and thus, the BP of the patients remains uncontrolled. Health24 News (2014) reported that only 10% of men and 18% of women with hypertension had their BP controlled at a level that would eliminate the risk of CVD complications. Poor control of hypertension continues, despite the implementation of physical exercise campaigns and programmes to curb a sedentary lifestyle for the control and the prevention of CVD complications. Vancampfort et al. (2017) also concluded that adults with chronic conditions are more likely to engage in less physical exercise. Therefore, the researcher asked the following question: What are the experiences of patients with hypertension at the PHC clinics in Soweto with regard to facilitating their own health-promoting lifestyle change measures such as regular physical exercise?

## Research purpose and objective

### Research purpose

This research article aims to explore and describe the experiences of patients with hypertension with regard to the facilitation of their own health-promoting lifestyle change measure of regular physical exercise.

### Research objective

The objective is to describe the experiences of patients with hypertension with regard to the facilitation of their own health-promoting lifestyle change measure of regular physical exercise.

## Context of the study

The context of the study consisted of the three PHC clinics in Soweto which the patients attend for their hypertension treatment and follow-up care.

The logic in qualitative research requires that the research findings should be interpreted in the context or setting in which the data were collected (Burns & Grove 2011; Fouché & Delport 2011).

## Definition of key concepts

### Regular physical exercise

In the context of this study, regular physical exercise means engaging in routine aerobic physical activity for about 10 to 30 min a day for about three times a week to improve patients’ cardiovascular health (Barrett, Darker & Hussey 2012).

### Health promotion

The Ottawa Charter’s (WHO 1986) definition of health promotion incorporates ‘the development of personal skills’ to enable people, who may be patients with hypertension, to have more control over their own health. Murray, Zentner and Yakimo (2009) highlight another dimension of the definition of health promotion, namely that of behaviour motivated by people’s desire to increase their [*own*] well-being and health potential. This notion of people’s desire to increase their own health incorporates the active participation of the patients with hypertension in the facilitation of their own health-promoting lifestyle change measure of engaging in regular physical exercise.

### Hypertension

Hypertension is a NCD, a cardiovascular chronic condition where there is increased pressure in the arteries, resulting in an elevated BP level of 140/90 or more (BP ≥ 140/90 mmHg) (Seedat & Rayner 2012). Hypertension is considered to be one of the chronic conditions of lifestyle, owing to the fact that one of the most common causes of hypertension, among others, is living an unhealthy lifestyle, such as a sedentary lifestyle, by not engaging in regular physical exercise.

### Primary health care clinic

A PHC clinic is a health service or clinic that is accessible, available and affordable to the community (WHO 1978). In this study, the PHC clinic refers to the three fixed Soweto Community Health Centres (SCHCs) where the study was conducted. The three clinics provide comprehensive PHC services, including services for the follow-up treatment and control of hypertension, provided by primary care nurse (PCN) practitioners and other relevant health care providers. Patients with hypertension are also interactive facilitators in the treatment and improvement of their own quality of health.

## Research design and method

A qualitative research design, which is exploratory, descriptive and contextual in nature, was used to achieve the purpose of the study (Burns & Grove 2011; Grove, Burns & Gray 2013; Parahoo 2014).

### Target population and sampling

The accessible population, which included all patients in the three PHC clinics in Soweto, in which permission was granted to conduct the study, were targeted (Burns & Grove 2011). A purposive sampling method was used to select patients with hypertension who had the rich information and the desired knowledge to facilitate their own health-promoting lifestyle change, in collaboration with PCN practitioners, with regard to regular physical exercise (Burns & Grove 2011; Creswell 2014; Streubert-Speziale & Carpenter 2007; Polit & Beck 2013). A total of 44 patients with hypertension from all three PHC clinics, who consented to participate in the study, were sampled. In all, 44 patients were sampled with a breakdown of 16, 14 and 14 patients, respectively, from each clinic. Two focus groups were conducted in each PHC clinic, making up a total of 30 patients of between 5 and 8 patients per focus group. The remaining 14 patients among whom individual interviews were conducted were six, five and three patients with hypertension from the three PHC clinics, respectively.

### Data collection method

Data were collected initially by conducting audio-taped focus group interviews and later by individual audio-taped interviews of patients with hypertension (Grove et al. 2013). The participants, who were patients with hypertension, exercised autonomy in giving informed consent to choose to participate in the study. A separate permission was requested from the participants to audiotape the interviews (Burns & Grove 2011; Grove et al. 2013; Streubert-Speziale & Carpenter 2007). A central question was posed to the participants: ‘As a patient with hypertension, how is it for you to live a healthy lifestyle, with regard to regular physical exercise?’

### Data analysis method

Both the researcher and the independent coder used Tesch’s open-coding method of data analysis (Creswell 2014). This method involved the coding of the collected data into segments to make sense of all the audio-recorded transcribed responses of the participants (Polit & Beck 2013). After coding the data, the themes and categories were described and identified. The involvement of an independent coder during data analysis strengthens the trustworthiness of the research findings.

The analysed data allowed the researcher to gain a deeper understanding of the meanings that the participants ascribed to their experiences as patients with hypertension with regard to the facilitation of their own health-promoting lifestyle change measure of regular physical exercise.

## Literature control

The research findings, which were the experiences of patients with hypertension with regard to regular physical exercise, were conceptualised and integrated into existing theoretical frameworks by means of literature control. The verification of the findings against literature also involved the process of embedding and linking the findings in and to larger theoretical perspectives (Burns & Grove 2011; Fouché & Delport 2011; Holloway & Wheeler 2010).

### Principle of respect and autonomy (recruitment procedure, informed consent and data protection)

The researcher ensured respect to all participants by ensuring autonomous choice, self-determination and decision-making. The participants either chose to participate in the study or not by means of giving informed consent. Permission for audio-taping the interviews was requested from the participants. The audiotapes were kept safe and are to be destroyed after 2 years of the research being published. The confidentiality of the participants during and after data collection was also ensured by not using the real names of patients throughout the study. The consent forms with names are under lock and key and only the researcher and study leaders have access to the documents.

### Principle of non-maleficence (potential hazards)

The research participants were not caused any physical, mental or spiritual harm or exploitation during and after the research study had been conducted.

### Principle of beneficence (potential benefit)

The researcher acted in the best interest of all research participants by ensuring the principle of beneficence. The researcher thus executed the study in such a way that it benefited and promoted the welfare of all patients with hypertension who participated in the study. The participants were able to communicate their challenges with regard to engaging in regular physical exercise.

### Principle of justice (potential benefit)

The researcher ensured that all patients with hypertension benefit from the outcome, which are strategies developed on the basis of the research findings. The strategies are to promote health-promoting lifestyle changes, including regular physical exercise. The strategies are reported in a separate paper.

## Trustworthiness

The trustworthiness of the study was ensured throughout the study by following Lincoln and Guba’s (1985) method of trustworthiness. This method involved the implementation of the trustworthiness measures of credibility, transferability, dependability and confirmability, as used in qualitative research.

### Credibility (truth value)

Credibility was ensured by means of several actions.

#### Prolonged engagement

During the process of data analysis, prolonged engagement was observed by engaging an external data transcriber and an independent coder. The researcher also ensured long-term immersion in the data during the transcription and data analysis process by listening to the audiotapes over and over again. The steps of Tesch’s open-coding method of data analysis, as described by Creswell (2014), were used.

#### Reflexivity

Reflexivity as a measure of credibility was achieved through the researcher’s engaging in critical self-reflection on own interaction, biases, preferences and preconceptions during data collection from the participants and also during data analysis (Grove et al. 2013).

#### Peer evaluation

Credibility was also ensured by enhancing the accuracy of the account of the study findings and research report by using peer and senior researchers to evaluate and review the relevance of the methodology followed in this study (Creswell 2014).

#### Use of external auditor or examiner

To further ensure the credibility of this study, three external examiners, one international and two national, were engaged. The three external examiners were not involved during the formative stage of the study and thus objectively audited the completed research report of the comprehensive study (Creswell 2014).

### Transferability (applicability)

The researcher ensured possible transferability to similar contexts, by providing thick description of the demographic data of the participants and their experiences, as well as of the research findings, with direct quotations from participants (Lincoln & Guba 1985).

### Dependability (consistency)

The researcher ensured dependability by conducting the study under supervision and with the continuous guidance of two experienced qualitative researchers. All measures employed to ensure credibility were also used to ensure dependability (Lincoln & Guba 1985) as explained above.

### Confirmability (neutrality)

The researcher ensured that the study reflects authentic, accurate and relevant findings that can be traced back to the data’s original sources by using direct citations as experienced by the participants (Botma et al. 2010; Holloway & Wheeler 2010).

## Findings and discussion

The aim of this article is to present the experiences of patients with hypertension with regard to the facilitation of their own health-promoting lifestyle change measure of regular physical exercise. The importance of the study was to use the findings of the study to develop strategies to facilitate health-promoting lifestyle changes, which included regular physical exercise, for patients with hypertension in collaboration with PCN practitioners at the PHC clinics in Soweto. The findings are discussed and presented as demographic data and data on the experiences of the participants, respectively.

### Demographic data of participants

Six focus groups (30 patients) and 14 individual interviews making a total of 44 patients with hypertension who gave consent to participate in the study were interviewed in the three PHC clinics in Soweto. A trained research assistant assisted participants who could not write or read with the completion of the demographic form. The demographic data findings are presented for the purpose of highlighting the personal characteristics of the participants of the study.

#### Gender of participants

The gender depiction of the total participants comprised 13 males and 31 females. Moosa et al. (2016) report that hypertension is higher among women. There is also evidence of a higher prevalence of CVD among African women as mentioned by Mohan et al. (2013).

#### Age of participants

All the participants aged above 20 years, the majority being between 41 and 80 years of age. The findings are in line with Mohan et al. (2013), who mention that there is evidence of age-adjusted prevalence of hypertension in urban populations. Zheng et al. (2012) in China also found that hypertension prevalence increases with age.

#### Length of time the participants have had hypertension

The time span for which the participants have been suffering from hypertension ranged from 1 year to more than 10 years, meeting the inclusive criteria. This indicated that most of the participants could significantly contribute to the study from their experiences of facilitating their own lifestyle changes of regular physical exercise.

### Experiences of participants

The research findings of the broader holistic research study showed two themes and the relevant sub-themes as illustrated in Table 1.

**TABLE 1:** Themes and categories of patients with hypertension experiences.

Themes	Sub-themes
Theme 1:	
Patients’ experiences of poor self-care as a challenge in facilitating their own health-promoting lifestyle changes	1a. Experiences of poor eating habits 1b. Experiences of inability to stop smoking 1c. Experiences of social consumption of alcohol 1d. Experiences of a lack of regular physical exercise
Theme 2:	
Patients’ experiences of family constraints as a challenge in facilitating their own health-promoting lifestyle changes	2a. Experiences of poor economic status 2b. Experiences of sharing meals with family members

*Source*: Magobe 2016

For the purpose of this research report, only findings on Theme 1 and the Sub-theme 1d of the study will be reported on as illustrated in Table 2.

**TABLE 2:** Findings: theme and sub-theme of participants’ experiences with regard to lack of regular physical exercise.

Theme:	Sub-theme:
Patients’ experiences of a challenge of	Patients’ experiences of
Poor self-care in facilitating own health-promoting lifestyle changes	Poor self-efficacy regarding regular physical exercise

*Source*: Magobe 2016

The theme and sub-theme presented in Table 2 represent the research findings as challenges or difficulties that patients with hypertension experienced in their endeavour to facilitate their own health-promoting lifestyle of engaging in regular physical exercise. The findings are now discussed in detail.

#### Theme: Patients’ experiences of poor self-care as a challenge in facilitating their own health-promoting lifestyle changes

Participants experienced that they had to confront the challenge of poor self-care. The challenge made it difficult for patients to facilitate their own health-promoting lifestyle change measure of regular physical exercise as a result of poor self-efficacy.

#### Sub-theme: Patients’ experiences of poor self-efficacy regarding regular physical exercise

Self-efficacy is an individual’s perception of their own ability to perform a certain task (Ashley 2007). In the context of this study and article, the task is the patients’ ability to facilitate their own health-promoting lifestyle change of engaging in regular physical exercise. Bandura (1997) further describes self-efficacy as a psycho-social theory that concerns itself with people’s belief in their capabilities to produce desired effects with their actions. For patients with hypertension, it is about being proactive in meeting the challenge of poor self-care and poor self-efficacy by engaging in regular physical exercise for the purpose of controlling their BP and improving hypertension (Mohan et al. 2013). The findings are further described, discussed and evidenced by the participant’s verbatim responses.

#### Experiences of lack of regular physical exercise

Findings showed that patients with hypertension, in the context of the Soweto PHC clinics, experienced a lack of regular physical exercise; this showed poor self-care as a result of poor self-efficacy. This was evident when the participants said:

‘I do try to exercise sometimes, but you know I get tired … and I will do it for a month only, then I just get tired. I am not lazy, but ….’ (Participant 1, Female, Patient)

Another participant related that:

‘… with me I don’t walk long distances at all because of my chest, I get a tight chest when I walk a distance and I start coughing.’ (Participant 2, Male, Patient)

These findings show that the participants had reasons for not engaging in regular physical exercise. St. Marie (2011) confirms these excuses or reasons by mentioning that some reasons, such as anxiety, can lead to fear of physical exertion during exercise, especially in people or patients who are already overweight. Such people become self-conscious and consider themselves to be under the extra-special scrutiny of others.

AlQuaiz and Tavel (2009) found that the lack of willpower to adhere to regular physical exercise was a great barrier among PHC clinic patients. This implies poor self-efficacy in continuing with new or already started good habits with regard to the health-promoting lifestyle change of regular physical exercise and other activities.

Literature also shows other reasons why patients do not engage in regular physical exercise, such as time limitations and lack of self-motivation (EDSA 2012; St. Marie 2011). Limited time more specifically is a reason used by employed people such as mothers with small children. Working mothers may not have a babysitter at home after normal office hours, and physical exercise facilities may lack child care resources. A busy home schedule before and after work may contribute to female patients not engaging in regular physical exercise as a result of lack of time. The reasons that male patients state, among others, include physical fatigue due to strenuous work-related activities.

One patient considered age to be the main reason for not engaging in regular physical exercise. This was obvious when the participant stated that:

‘I don’t gym at all, I even refuse to walk with a friend when invited, my knees are not strong enough for walking and my age also … I am 52 years old now.’ (Participant 3, Female, Patient)

These findings evidently show that some people or patients with hypertension will not engage in physical exercise, even if it is to improve their health. The organisation of EDSA acknowledges that some people just hate to exercise (EDSA 2012). In order to realise the related health benefits, patients with hypertension should engage in regular physical exercise, as advocated by Stuart-Shor et al. (2012) and Strydom (2013); the health benefits of regular physical exercise include improved circulation to the heart, lungs and muscles, improved muscle tone and lessened joint stiffness in the elderly. BP is also effectively reduced and hypertension controlled when a patient engages in regular physical exercise owing to a strong link between health and physical exercise (SRSA 2009; Strydom 2013).

Other benefits of regular physical exercise for patients with hypertension include a reduction in stress, meaning an improvement in mental health, including improved appetite and a subsequent improvement in quality of life (Strydom 2013; Stuart-Shor et al. 2012). People who do not engage in regular physical exercise have a higher risk of being overweight or obese, which is a major added risk factor for cardiovascular complications such as stroke and coronary artery disease, as mentioned in Sport and Recreation South Africa (SRSA 2009) and also by Stuart-Shor et al. (2012). It is further maintained that addressing obesity through physical exercise is also critical for reducing overall CVD risk (Stuart-Shor et al. 2012).

### Ethical considerations

The following ethical considerations, based on the four principles of health care practice, as explained by Dhai and McQuoid-Mason (2011), were adhered to in this study: the principle of respect and autonomy, the principle of non-maleficence, the principle of beneficence and the principle of justice.

## Conclusion

Lifestyle modification remains the first step treatment for all stages of hypertension, which includes regular physical exercise and its health benefits. Findings in this study, however, show that participants experienced lack of regular physical exercise, showing poor self-care due to poor self-efficacy. All people have the personal quality of self-efficacy, which can be effectively developed (Bandura 1997). PCN practitioners should, therefore, facilitate the unfolding and development of hypertensive patients’ self-efficacy. This can be achieved by continuously educating, motivating and empowering patients with the necessary knowledge, skill and the values of self-management during follow-up care at the PHC clinics in Soweto. Without self-efficacy, patients are not able to positively make daily self-management decisions in the process of facilitating their own health-promoting lifestyle change of regular physical exercise. Engaging in regular physical exercise will also help in the implementation of the strategic objective of the NSRP, which is to improve the health and well-being of the South African population (SRSA 2012).
